# Combined Effects of *ICAM-1* Single-Nucleotide Polymorphisms and Environmental Carcinogens on Oral Cancer Susceptibility and Clinicopathologic Development

**DOI:** 10.1371/journal.pone.0072940

**Published:** 2013-09-12

**Authors:** Chiao-Wen Lin, Chun-Yi Chuang, Chih-Hsin Tang, Junn-Liang Chang, Liang-Ming Lee, Wei-Jiunn Lee, Jyh-Ming Chow, Shun-Fa Yang, Ming-Hsien Chien

**Affiliations:** 1 Institute of Oral Sciences, Chung Shan Medical University, Taichung, Taiwan; 2 Department of Dentistry, Chung Shan Medical University Hospital, Taichung, Taiwan; 3 School of Medicine, Chung Shan Medical University, Taichung, Taiwan; 4 Department of Otolaryngology, Chung Shan Medical University Hospital, Taichung, Taiwan; 5 Graduate Institute of Basic Medical Science, China Medical University, Taichung, Taiwan; 6 Department of Biotechnology, College of Health Science, Asia University, Taichung, Taiwan; 7 Department of Medical Management, Taoyuan Armed Forces General Hospital, Taoyuan County, Taiwan; 8 School of Medicine, Pathology Department, National Defense Medical Center, Taipei, Taiwan; 9 Department of Urology, Wan Fan Hospital, Taipei Medical University, Taipei, Taiwan; 10 Section of Hematology-Oncology, Department of Internal Medicine, Wan Fang Hospital, Taipei Medical University, Taipei, Taiwan; 11 Institute of Medicine, Chung Shan Medical University, Taichung, Taiwan; 12 Department of Medical Research, Chung Shan Medical University Hospital, Taichung, Taiwan; 13 Wan Fang Hospital, Taipei Medical University, Taipei, Taiwan; 14 Graduate Institute of Clinical Medicine, College of Medicine, Taipei Medical University, Taipei, Taiwan; The University of Hong Kong, China

## Abstract

**Background:**

In Taiwan, oral cancer has causally been associated with environmental carcinogens. Intercellular adhesion molecule (ICAM)-1, a cell adhesion molecule with a key role in inflammation and immunosurveillance, was implicated in carcinogenesis by facilitating instability in the tumor environment. The current study explored the combined effect of *ICAM-1* gene polymorphisms and exposure to environmental carcinogens on the susceptibility of developing oral squamous cell carcinoma (OSCC) and the clinicopathological characteristics of the tumors.

**Methodology and Principal Findings:**

Four single-nucleotide polymorphisms (SNPs) of the *ICAM-1* gene from 595 patients with oral cancer and 561 non-cancer controls were analyzed by a real-time PCR. We found that the *ICAM-1* rs5498 polymorphism and the TAGG or TACG haplotype of 4 *ICAM-1* SNPs (rs3093030, rs5491, rs281432, and rs5498) combined were associated with oral-cancer susceptibility. Among 727 smokers, *ICAM-1* polymorphisms carriers with the betel-nut chewing habit had a 27.49–36.23-fold greater risk of having oral cancer compared to *ICAM-1* wild-type (WT) carriers without the betel-nut chewing habit. Among 549 betel-nut chewers, *ICAM-1* polymorphisms carriers who smoked had a 9.93–14.27-fold greater risk of having oral cancer compared to those who carried the WT but did not smoke. Finally, patients with oral cancer who had at least 1 T allele of *ICAM-1* rs5491 or 1 G allele of rs281432 were at lower risk of developing an advanced clinical stage (III/IV) (*p*<0.05), compared to those patients with AA or CC homozygotes.

**Conclusions:**

Our results suggest that the *ICAM-1* rs5498 SNP and either of 2 haplotypes of 4 SNPs combined have potential predictive significance in oral carcinogenesis. Gene-environment interactions of *ICAM-1* polymorphisms, smoking, and betel-nut chewing might alter oral-cancer susceptibility. *ICAM-1* rs5491 and rs281432 may be applied as factors to predict the clinical stage in OSCC patients.

## Introduction

Oral cancers can originate in any tissues of the mouth, but approximately 90% are squamous cell carcinomas (SCCs) [1]. Such cancers are known worldwide for their poor prognosis and major oncologic problems. In Taiwanese males, oral cancer is ranked as the fourth most common type of cancer, with a peak at 55–59 years old, and is the leading type of cancer causing death in the 40–50-year-old age group [2].

Oral SCC (OSCC) development is a multistep process requiring the accumulation of multiple genetic alterations, influenced by a patient's genetic predisposition and by environmental factors, which include alcohol and tobacco consumption, betel-nut chewing, chronic inflammation, and viral infection [3]–[6]. Among genetic factors, single-nucleotide polymorphisms (SNPs) are the most common type of DNA sequence variation which influences the occurrence and progression of gene-related diseases. Previous reports showed that SNPs may possibly predict the risk of oral cancer [7]–[9]. Moreover, combinations of environmental carcinogens and certain gene polymorphisms might also increase a person's susceptibility to oral cancer [7]–[9]. Thus, to elucidate the complex process of carcinogenesis and improve the scientific basis for preventive interventions, the identification of major genes influencing a patient's susceptibility to OSCC should be prioritized.

Intercellular adhesion molecule (ICAM)-1, also known as CD54, is a transmembrane glycoprotein in the immunoglobulin (Ig) superfamily containing five extracellular Ig-like domains, a transmembrane domain, and a short cytoplasmic tail [10]–[14]. Recently, it was shown that ICAM-1 possibly contributes to tumorigenesis and metastasis including oral cancer [15]–[17]. Binding of tumor cells to endothelial ICAM-1 [18] leads to auto-upregulation of tumor ICAM-1 and more importantly to chemotaxis of tumor-associated macrophages and neutrophils that eventually facilitate loosening of adhesive contacts and the breaking down of endovascular barriers, permitting tumor cell migration, neoangiogenesis, and ultimately instability of the tumor environment [15]–[17]. This mechanism is supported by studies showing that patients with increased ICAM-1 expression in tumors have more-advanced stages of the disease [15], [19].

ICAM-1 also exists in a soluble form (sICAM-1) which proteolytically cleaves the full-length ICAM-1 near its transmembrane region. sICAM-1 is partially detectable in the serum of healthy subjects, but its level is elevated with inflammatory and malignant disorders [20]–[22]. The positive correlation of the sICAM-1 serum level and clinical tumor size/lymph node involvement/metastasis staging of some human malignancies was reported [23]–[25]. The main cause of sICAM-1 release in human malignancies is not well defined; but in recent studies, matrix metalloproteinase (MMP)-9 and human leukocyte elastase were implicated in this process [22], [26], [27].

Prior research reported that polymorphic variations in exon (rs5498 and rs5491) or intron (rs281432) regions of the *ICAM-1* gene and in the region (rs3093030) between the *ICAM-1* and *ICAM-4* genes were associated with risks for prostate cancer, gastric cancer, breast cancer, type 1 diabetes, metabolic syndrome, and systemic lupus erythematosus [28]–[33]. Until now, to the best of our knowledge, there has been no documented report studying these polymorphisms in oral cancer. The current study investigated relationships of SNPs (rs3093030, rs5498, rs5491, and rs281432) of the *ICAM-1* gene with the risk of oral cancer. The influences of these SNPs combined with betel-nut and tobacco consumption, leading to susceptibility to oral cancer, were evaluated. We also investigated the relationship between genetic influences and the clinicopathological characteristics of oral cancer.

## Materials and Methods

### Subjects and Specimen Collection

In 2007–2012, we recruited 595 patients (573 males and 22 females with a mean age of 54.36±11.31 years) at Chung Shan Medical University Hospital in Taichung and Changhua Christian Hospital and Show Chwan Memorial Hospital in Changhua, Taiwan as the case group. Meanwhile, controls were enrolled from the physical examination during those three hospitals, which are also the facilities that cases were collected from. At the end of recruitment, a total of 561 non-cancer participants that had neither self-reported history of cancer of any sites were included. In addition, subjects with oral precancerous disease such as oral submucous fibrosis, leukoplakia, erythroplakia, verrucous hyperplasia, etc. were excluded from control group. For both cases and controls, we used a questionnaire to obtain exposure information about betel-nut chewing, tobacco use, and alcohol consumption. Medical information of the cases, including TNM clinical staging, the primary tumor size, lymph node involvement, and histologic grade, was obtained from their medical records. Oral-cancer patients were clinically staged at the time of their diagnosis according to the TNM staging system of the American Joint Committee on Cancer (AJCC) Staging Manual (7th ed.). Tumor differentiation was examined by a pathologist according to the AJCC classification. Whole-blood specimens collected from controls and OSCC patients were placed in tubes containing ethylenediaminetetraacetic acid (EDTA), were immediately centrifuged, and then stored at −80°C. This study was approved by the Institutional Review Boards of Show Chwan Memorial Hospital, and informed written consent to participate in the study was obtained from each individual.

### Selection of ICAM-1 Polymorphisms

In the dbSNP database, over 20 SNPs have been documented in the 7-exon region of the ICAM-1 gene. We included the non-synonymous SNPs rs5491 (K56M in exon 2) and rs5498 (E469K in exon 6) in the coding sequences of the gene. To obtain adequate power to evaluate the potential association, we investigated rs281432 (C/G in intron 2) with minor allelic frequencies of >5%. Furthermore, another SNP between the ICAM-1 and ICAM-4 genes (rs3093030) was selected in this study since this SNP was found to affect the production of sICAM-1 in a Chinese population [34].

### Genomic DNA Extraction

Genomic DNA was extracted using QIAamp DNA blood mini kits (Qiagen, Valencia, CA, USA) following the manufacturer's instructions [35]. We dissolved DNA in TE buffer (10 mM Tris and 1 mM EDTA; pH 7.8) and then quantified it by measuring the optical density at 260 nm. The final preparation was stored at −20°C and used to create templates for the polymerase chain reaction (PCR) [36].

### Real-time PCR

Allelic discrimination of the rs3093030, rs5498, rs5491, and rs281432 polymorphisms of the *ICAM-1* gene was assessed with the ABI StepOne™ Real-Time PCR System (Applied Biosystems, Foster City, CA, USA), and analyzed with SDS vers. 3.0 software (Applied Biosystems) using the TaqMan assay [37]. The primer sequences and probes for analysis of the *ICAM-1* gene polymorphisms are described in Table 1. The final volume for each reaction was 5 µL, containing 2.5 µL TaqMan Genotyping Master Mix, 0.125 µL TaqMan probe mix, and 10 ng genomic DNA. The real-time PCR included an initial denaturation step at 95°C for 10 min, followed by 40 cycles at of 95°C for 15 s and then at 60°C for 1 min. For each assay, appropriate controls (nontemplate and known genotype) were included in each typing run to monitor reagent contamination and as a quality control. To validate results from real-time PCR, around 5% of assays were repeated and several cases of each genotype were confirmed by the DNA sequence analysis.

**Table 1:** TaqMan primer sets for *ICAM-1* genotyped SNPs.

SNP	Probe
*ICAM-1* rs3093030	VIC-5′- TTGTGGGTTGATGGCCATACC
	FAM-5′- ATTGTGGGTTGATGGTCATACC
*ICAM-1* rs5491	VIC-5′- TCCTGTGACCAGCCCAAGTTGT
	FAM-5′- CCTGTGACCAGCCCATGTTGT
*ICAM-1* rs281432	VIC-5′- TGGAGGGTTTCTGAGCAGG
	FAM-5′- TGGAGGGTTTGTGAGCAGG
*ICAM-1* rs5498	VIC-5′- AGGTCACCCGCAAGGTGAC
	FAM-5′- AGGTCACCCGCGAGGTGAC

### Statistical Analysis

Differences between the 2 groups were considered significant if *p* values were <0.05. Hardy-Weinberg equilibrium (HWE) was assessed using a goodness-of-fit *X^2^*-test for biallelic markers. The Mann-Whitney *U*-test and Fisher's exact test were used to compare differences in the distributions of patient demographic characteristics between the non-cancer (control) and oral-cancer groups. The adjusted odds ratios (ORs) and 95% confidence intervals (CIs) of the association between genotype frequencies and risk plus clinicopathological characteristics were estimated using multiple logistic regression models, after controlling for other covariates. We analyzed all data with Statistical Analytic System (SAS Institute, Cary, NC, USA) software (vers. 9.1, 2005) for Windows.

## Results

Results of the statistical analysis of demographic characteristics are shown in Table 2. We found significantly different distributions of age (*p* = 0.001), gender (*p*<0.0001), betel-nut chewing (*p*<0.0001), alcohol consumption (*p*<0.0001), and tobacco use (*p*<0.0001) between control participants and OSCC patients. To diminish the possible interference of environmental factors, adjusted ORs (AORs) with 95% CIs were estimated by multiple logistic regression models after controlling for other covariates in each comparison.

**Table 2:** The distributions of demographical characteristics in 561 controls and 595 patients with oral cancer.

Variable	Controls (N = 561)	Patients (N = 595)	OR (95% CI)	*p* value
**Age (yrs)**	**Mean ± S.D.**	**Mean ± S.D.**		
	51.81±14.71	54.36±11.31		*p* = 0.001*
**Gender**	**n (%)**	**n (%)**		
Male	457 (81.5%)	573 (96.3%)		
Female	104 (18.5%)	22 (3.7%)		*p*<0.0001*
**Betel nut chewing**				
No	468 (83.4%)	139 (23.4%)	Reference	
Yes	93 (16.6%)	456 (76.6%)	16.509 (12.322–22.119)	*p*<0.0001*
**Alcohol consumption**				
No	347 (61.9%)	243 (40.8%)	Reference	
Yes	214 (38.1%)	352 (59.2%)	2.349 (1.855–2.974)	*p*<0.0001*
**Tobacco consumption**				
No	341 (60.8%)	88 (14.8%)	Reference	
Yes	220 (39.2%)	507 (85.2%)	8.930 (6.731–11.848)	*p*<0.0001*

Mann-Whitney U test or Fisher's exact test was used between healthy controls and patients with oral cancer.

* *p* value<0.05 as statistically significant.

In our recruited control group, frequencies of *ICAM-1* genes were in HWE (*p*>0.05). Genotype distributions and associations between oral cancer and *ICAM-1* gene polymorphisms are shown in Table 3. Alleles with the highest distribution frequency for the rs3093030, rs5491, rs281432, and rs5498 genes of *ICAM-1* in both of our recruited oral-cancer patients and healthy controls were respectively homozygous for C/C, homozygous for A/A, homozygous for C/C, and homozygous for A/A. After adjusting for several variables, there were no significant differences in the incidences of oral cancer in individuals with the rs3093030, rs5491, and rs281432 polymorphisms of the *ICAM-1* gene compared to wild-type (WT) individuals. However, subjects with the *ICAM-1* polymorphic rs5498 AG, GG, and the combination of AG and GG genotypes respectively exhibited significantly (*p*<0.05) higher risks of 1.377- (95% CI: 1.006–1.968), 1.202- (95% CI: 1.029–4.712), and 1.465-fold (95% CI: 1.041–2.063) of having OSCC compared to their corresponding WT homozygotes.

**Table 3:** Distribution frequency of *ICAM-1* genotypes in 561 healthy controls and 595 oral cancer patients.

Variable	Controls (N = 561) n (%)	Patients (N = 595) n (%)	OR (95% CI)	AOR (95% CI)
**rs3093030**				
CC	365 (65.1%)	384 (64.5%)	1.00	1.00
CT	179 (31.9%)	183 (30.8%)	0.972 (0.756–1.249)	1.126 (0.784–1.617)
TT	17 (3.0%)	28 (4.7%)	1.566 (0.843–2.909)	1.149 (0.468–2.822)
CT+TT	196 (34.9%)	211 (35.5%)	1.023 (0.804–1.303)	1.129 (0.797–1.599)
**rs5491**				
AA	514 (91.6%)	537 (90.3%)	1.00	1.00
AT	47 (8.4%)	55 (9.2%)	1.120 (0.745–1.684)	1.523 (0.826–2.809)
TT	0 (0%)	3 (0.5%)	—	—
AT+TT	47 (8.4%)	58 (9.7%)	1.181 (0.789–1.768)	1.618 (0.887–2.953)
**rs281432**				
CC	324 (57.8%)	332 (55.8%)	1.00	1.00
CG	200 (35.7%)	218 (36.6%)	1.064 (0.832–1.360)	1.211 (0.849–1.728)
GG	37 (6.6%)	45 (7.6%)	1.187 (0.748–1.882)	1.199 (0.604–2.379)
CG+GG	237 (42.2%)	263 (44.2%)	1.083 (0.858–1.367)	1.209 (0.863–1.696)
**rs5498**				
AA	350 (62.4%)	329 (55.3%)	1.00	1.00
AG	182 (32.4%)	220 (37.0%)	**1.286 (1.004–1.647)** *	**1.377 (1.006–1.968)** *
GG	29 (5.2%)	46 (7.7%)	**1.687 (1.035–2.750)** *	**1.202 (1.029–4.712)** *
AG+GG	211 (37.6%)	266 (44.7%)	**1.341 (1.060–1.697)** *	**1.465 (1.041–2.063)** *

The odds ratios (ORs) and with their 95% confidence intervals (CIs) were estimated by logistic regression models. The adjusted odds ratios (AORs) with their 95% confidence intervals (CIs) were estimated by multiple logistic regression models after controlling for age, gender, betel nut chewing, tobacco and alcohol consumption.

* *P* value<0.05 as statistically significant.

Interactive effects between environmental risk factors and genetic polymorphisms of *ICAM-1* are shown in Tables 4 and 5. Among 727 smokers, subjects with at least 1 T allele of rs3093030 or rs5491, 1 G allele of rs281432 or rs5498, and the betel-nut chewing habit respectively had 35.47- (95% CI: 16.497–76.242), 32.49- (95% CI: 8.325–126.775), 36.233- (95% CI: 17.596–74.610), and 27.49-fold (95% CI: 13.856–54.553) higher risks of having oral cancer. Individuals with either at least 1 T allele of rs3093030 or rs5491, 1 G allele of rs281432 or rs5498, or who chewed betel nut had respective 7.51- (95% CI: 4.522–12.486), 18.43- (95% CI: 11.093–30.620), 11.35- (95% CI: 6.842–19.861), and 7.49-fold (95% CI: 4.403–12.756) higher risks of having oral cancer compared to individuals with WT homozygotes who did not chew betel- nut (Table 4).

**Table 4:** Adjusted odds ratio (AOR) and 95% confidence interval (CI) of oral cancer associated with *ICAM-1* genotypic frequencies and betel nut chewing among 727 smokers.

Variable	Controls (n = 220) (%)	Patients (n = 507) (%)	OR (95% CI)	AOR (95% CI)
**rs3093030**				
aCC genotype & non-betel nut chewing	98 (44.5%)	54 (10.7%)	1.00	1.00
bCT or TT genotype or betel nut chewing	105 (47.7%)	300 (59.2%)	**5.185 (3.477–7.733)**	**7.514 (4.522–12.486)**
cCT or TT genotype with betel nut chewing	17 (7.8%)	153 (30.1%)	**16.333 (8.953–29.796)**	**35.465 (16.497–76.242)**
**rs5491**				
aAA genotype & non-betel nut chewing	141 (64.1%)	67 (13.2%)	1.00	1.00
bAT or TT genotype or betel nut chewing	76 (34.5%)	403 (79.5%)	**11.159 (7.629–16.324)**	**18.430 (11.093–30.620)**
cAT or TT genotype with betel nut chewing	3 (1.4%)	37 (7.3%)	**29.955 (7.724–87.213)**	**32.487 (8.325–126.775)**
**rs281432**				
aCC genotype & non-betel nut chewing	99 (45.0%)	42 (8.3%)	1.00	1.00
bCG or GG genotype or betel nut chewing	99 (45.0%)	281 (55.4%)	**6.690 (4.363–10.259)**	**11.346 (6.482–19.861)**
cCG or GG genotype with betel nut chewing	22 (10.0%)	184 (36.3%)	**19.714 (11.141–34.886)**	**36.233 (17.596–74.610)**
**rs5498**				
aAA genotype & non-betel nut chewing	92 (41.8%)	44 (8.7%)	1.00	1.00
bAG or GG genotype or betel nut chewing	103 (46.8%)	274 (54.0%)	**5.562 (3.637–8.505)**	**7.493 (4.403–12.756)**
cAG or GG genotype with betel nut chewing	25 (11.4%)	189 (37.3%)	**15.807 (9.115–27.412)**	**27.493 (13.856–54.553)**

The odds ratios (ORs) with their 95% confidence intervals were estimated by logistic regression models.

The adjusted odds ratios (AORs) with their 95% confidence intervals were estimated by multiple logistic regression models after controlling for age, gender and alcohol consumption.

a Individual with wild genotype but without betel nut chewing.

b Individual with either at least one mutated genotype or betel nut chewing.

c Individual with both at least one mutated genotype and betel nut chewing.

**Table 5:** Adjusted odds ratio (AOR) and 95% confidence interval (CI) of oral cancer associated with *ICAM-1* genotypic frequencies and smokers among 549 betel nut consumers.

Variable	Controls (n = 93) (%)	Patients (n = 456) (%)	OR (95% CI)	AOR (95% CI)
**rs3093030**				
aCC genotype & non-smoker	15 (16.1%)	17 (3.7%)	1.00	1.00
bCT or TT genotype or smoker	61 (65.6%)	286 (62.7%)	**4.137 (1.959–8.734)**	**4.173 (1.672–10.416)**
cCT or TT genotype with smoking	17 (18.3%)	153 (33.6%)	**7.941 (3.373–18.696)**	**11.992 (4.134–34.785)**
**rs5491**				
aAA genotype & non-smoker	19 (20.4%)	21 (4.6%)	1.00	1.00
bAT or TT genotype or smoker	71 (76.3%)	398 (87.3%)	**5.072 (2.595–9.911)**	**8.016 (3.303–19.454)**
cAT or TT genotype with smoking	3 (3.2%)	37 (8.1%)	**11.159 (2.951–42.200)**	**14.273 (2.851–71.452)**
**rs281432**				
aCC genotype & non-smoker	15 (16.1%)	13 (2.9%)	1.00	1.00
bCG or GG genotype or smoker	56 (60.2%)	259 (56.8%)	**5.337 (2.405–11.840)**	**8.063 (2.901–22.413)**
cCG or GG genotype with smoking	22 (23.7%)	184 (40.4%)	**9.650 (4.066–22.905)**	**13.765 (4.586–41.318)**
**rs5498**				
aAA genotype & non-smoker	15 (16.1%)	12 (2.6%)	1.00	1.00
bAG or GG genotype or smoker	53 (57.0%)	255 (55.9%)	**6.014 (2.663–13.583)**	**5.723 (2.150–15.232)**
cAG or GG genotype with smoking	25 (26.9%)	189 (41.4%)	**9.450 (3.974–22.469)**	**9.933 (3.516–28.058)**

The odds ratios (ORs) with their 95% confidence intervals were estimated by logistic regression models.

The adjusted odds ratios (AORs) with their 95% confidence intervals were estimated by multiple logistic regression models after controlling for age, gender and alcohol consumption.

a Individual with wild genotype but without smoking.

b Individual with either at least one mutated genotype or smoking.

c Individual with both at least one mutated genotype and smoking.

Among betel-nut consumers in our cohort, subjects with *ICAM-1* polymorphic rs3093030, rs5491, rs281432, or rs5498 genes and who smoked had corresponding 11.99- (95% CI: 4.134–34.785), 14.27- (95% CI: 2.851–71.452), 13.77- (95% CI: 4.586–41.318), and 9.93-fold (95% CI: 3.516–28.058) higher risks of having oral cancer compared to betel-nut chewers with the WT gene who did not smoke (Table 5). Moreover, people who were either polymorphic for *ICAM-1* in 4 loci (rs3093030, rs5491, rs281432, and rs5498) or who smoked were at a 4.17∼8.06-fold risk (*p*<0.05) of developing oral cancer, compared to people with the WT gene who did not smoke (Table 5). In light of the above results, we suggest that *ICAM-1* gene polymorphisms have strong impacts on oral-cancer susceptibility in betel-nut and/or smoking consumers.

To explore the effects of polymorphic genotypes of *ICAM-1* on the clinical status of OSCC, we classified OSCC patients into 2 subgroups. In the first subgroup, patients had homozygous WT alleles; in the other subgroup they had at least 1 polymorphic allele. For the genotypic frequencies of the SNPs, only *ICAM-1* rs5491 and rs281432 showed significant associations with clinical pathological variables in OSCC patients. Compared to the WT genotype, patients with at least 1 polymorphic T allele of *ICAM-1* rs5491 or 1 polymorphic G allele of *ICAM-1* rs281432 showed a significant lower risk (*p*<0.05) for being at an advanced clinical stage (III/IV) (Table 6).

**Table 6:** Clinical status and *ICAM-1* genotypic frequencies in 595 oral cancer patients.

Variable	rs3093030			rs5491			rs281432			rs5498		
	CC (N = 384)	CT+TT (N = 211)	*p* value	AA (N = 537)	AT+TT (N = 58)	*p* value	CC (N = 332)	CG+GG (N = 263)	*p* value	AA (N = 329)	AG+GG (N = 266)	*p* value
**Clinical Stage**												
Stage I/II	168 (43.8%)	93 (44.1%)	0.939	226 (42.1%)	35 (60.3%)	**0.008** *	131 (39.5%)	130 (49.4%)	**0.015** *	136 (41.3%)	125 (47.0%)	0.167
Stage III/IV	216 (56.2%)	118 (55.9%)		311 (57.9%)	23 (39.7%)		201 (60.5%)	133 (50.6%)		193 (58.7%)	141 (53.0%)	
**Tumor size**												
≤T2	239 (62.2%)	124 (58.8%)	0.406	322 (60.0%)	41 (70.7%)	0.112	193 (58.1%)	170 (64.6%)	0.106	195 (59.3%)	168 (63.2%)	0.334
>T2	145 (37.8%)	87 (41.2%)		215 (40.0%)	17 (29.3%)		139 (41.9%)	93 (35.4%)		134 (40.7%)	98 (36.8%)	
**Lymph node metastasis**												
No	242 (63.0%)	137 (64.9%)	0.643	337 (62.8%)	42 (72.4%)	0.146	208 (62.7%)	171 (65.0%)	0.551	206 (62.6%)	173 (65.0%)	0.541
Yes	142 (37.0%)	74 (35.1%)		200 (37.2%)	16 (27.6%)		124 (37.3%)	92 (35.0%)		123 (37.4%)	93 (35.0%)	
**Distant metastasis**												
No	378 (98.7%)	209 (98.5%)	0.533	529 (98.5%)	58 (100.0%)	0.349	325 (97.9%)	262 (99.6%)	0.069	322 (97.9%)	35 (99.6%)	0.065
Yes	6 (1.6%)	2 (0.9%)		8 (1.5%)	0 (0%)		7 (2.1%)	1 (0.4%)		7 (2.1%)	1 (0.4%)	

T2: tumor size >2 cm in the greatest dimension.

* P value<0.05 as statistically significant.

We further explored the haplotypes to evaluate the combined effect of the 4 polymorphisms on oral-cancer susceptibility. The distribution frequencies of the *ICAM-1* rs3093030, rs5491, rs281432, and rs5498 haplotypes in our recruited individuals were analyzed. Three haplotypes had frequencies of >5% among all cases; the most common haplotype in the control was CACA (71.6%); and it was therefore chosen as a reference. Compared to the reference, 2 *ICAM-1* haplotypes, TAGG and TACG, significantly (*p*<0.05) respectively increased the risks for OSCC by 1.69- (95% CI: 1.256–2.283) and 1.45-fold (95% CI: 1.028–2.043) (Table 7).

**Table 7:** Distribution frequency of *ICAM-1* haplotype in controls and oral cancer patients.

Variable				Controls (N = 1122) n (%)	Patients (N = 1190) n (%)	OR (95% CI)	*P* value
rs3093030	rs5491	rs281432	rs5498				
C/T	A/T	C/G	A/G				
C	A	C	A	803 (71.6%)	772 (64.9%)	Reference	
T	A	G	G	78 (7.0%)	127 (10.7%)	**1.694 (1.256–2.283)**	**<0.001**
T	A	C	G	61 (5.4%)	85 (7.1%)	**1.449 (1.028–2.043)**	**0.033**
Other#				180 (16.0%)	206 (17.3%)	1.190 (0.952–1.488)	0.125

# Others: CAGA (54),CACG (53), CTGG (26), TAGA(23), CAGG(19), CTGA(3), TACA (2).

## Discussion

ICAM-1 is believed to play an important role in several malignancies. In breast, gastric, and colorectal cancers, increased ICAM-1 expression in cancer cells was correlated with a more-favorable prognosis, suggesting a role of ICAM-1 in enhancement of immune surveillance [38]–[40]. Conversely, the potential involvement of ICAM-1 expression in cancer invasion and metastasis was reported in melanomas, and pancreatic, lung, and oral cancers [15], [17], [41]. Thus, the biological significance of ICAM-1 expression in cancer remains controversial. In this study, we first investigated whether polymorphisms within the *ICAM-1* gene likely played a significant role in the susceptibility to and development of oral cancer. Four SNPs were selected for inclusion in this hypothesis-generating study, two of which (rs5491 and rs5498) encode amino acid substitutions in the expressed ICAM-1 molecule and all of which are thought to affect production of sICAM-1 in Chinese populations [31], [34]. Our data showed that individuals with the *ICAM-1* rs5498-G allele had a higher risk for OSCC compared to the WT genotype. Similar to our results, the *ICAM-1* rs5498 SNP showed a statistical difference in susceptibility to prostate cancer [28], breast cancer [33], and grade II astrocytomas [42].

The rs5498 polymorphism entailed a glutamic acid (rs5498 A/G or G/G) to lysine (rs5498 A/A) substitution within coding exon 6 that was thought to lead to decreased integrin receptor binding and increased sICAM-1 production in a German pediatric asthma case-control study [43]. The amino-acid exchange between glutamic acid (negative polar) and lysine (positive polar) is located in the fifth Ig-like domain of ICAM-1. This region seems to be particularly important for the dimerization of ICAM-1. Compared to ICAM-1 monomers, ICAM-1 dimers exhibit enhanced binding to lymphocyte function-associated protein-1 [44]. Therefore, the amino-acid exchange might diminish ICAM-1 dimerization and in turn lead to decreased integrin receptor binding. Moreover, sICAM-1 lacks transmembrane and intracellular domains, which are crucial for efficient transendothelial adhesion [45] and migration of lymphocytes [46]. As it is soluble, it may compete with membrane ICAM-1 for β2-integrin binding, hence further inhibiting leukocyte recruitment trafficking [47]. Taken together, it appears that within an rs5498 AG or GG genetic environment, the increased incidence of OSCC might be due to defects in leukocyte functions, which can ultimately lead to defective immunosurveillance, shortened cancer dormancy, stimulation of angiogenesis, and growth of tumor cells. In contrast to rs5498, we also found that oral-cancer patients with 1 T allele of *ICAM-1* rs5491 or 1 G allele of *ICAM-1* rs281432 showed a significant lower risk for being at an advanced clinical stage. Thus, these 2 SNPs might confer protection against the progression of OSCC, but the underlying mechanisms of polymorphic rs5491 and rs281432 on OSCC progression are still unknown. Further specifically designed studies are needed to verify the effects and underlying mechanism of polymorphic rs5498, rs5491, and rs281432 on OSCC progression. For example, we should first determine the correlation between sICAM-1 levels and rs5498, rs5491, or rs281432 SNPs in OSCC patients.

Alcohol consumption, tobacco smoking, and betel-nut chewing are the main known etiologic factors for oral cancer. In this study, higher ratios were observed of individuals who had chewed betel nut and consumed alcohol and tobacco in the group of OSCC patients (76.6%, 59.2%, and 85.2%, respectively) than control subjects (16.6%, 38.1%, and 39.2%, respectively), which indicates that these 3 environmental carcinogens may be associated with risks for oral cancer. Betel-nut and tobacco consumption appeared to be particularly strongly associated with oral cancer, a finding that is congruent with prior research [6]. Betel-nut chewing was found to stimulate the protein level of matrix metalloproteinase (MMP)-9 in the saliva of healthy people [48]. Lime-piper betel nut may also increase protein levels of the c-fos and c-jun proto-oncogenes [49]. Consumption of tobacco may significantly induce the expression of nuclear hypoxia-inducible factor (HIF)-1α, which is an unfavorable prognostic factor in oral cancer [50]. Moreover, cigarette smoke condensate was also found to induce MMP-9 expression in oral keratinocytes [51]. These lines of evidence suggest that environmental carcinogen exposure is involved in the formation or pathogenesis of oral cancer.

Exposure to environmental carcinogens might partially involve the formation or pathogenesis of oral cancer, but increasing evidence indicates that genomic changes progressively alter cellular phenotypes and might more significantly lead cells to evolve from the preneoplastic stage into cancer [52]. In our study, only *ICAM-1* rs5498 SNPs alone contributed to oral-cancer susceptibility (Table 3). The synergistic effects of environmental factors (betel-nut and smoking) and 4 *ICAM-1* gene SNPs (rs3093030, rs5491, rs281432, and rs5498) on the risk of oral cancer (Tables 4 and 5) are well demonstrated. Betel-nut and tobacco carcinogens might enhance MMP-9 expression, and then alter the proteolytic cleavage of the full-length ICAM-1 [27]. Consequently, it may upregulate sICAM-1 to affect immunosurveillance and then promote oral cancer formation.

A variety of SNPs might be silent, that is to say, with no direct effect on gene products. However, by virtue of linkage disequilibrium (LD) that exists across the human genome, they can still be used as genetic markers to locate adjacent functional variants that contribute to disease. When each SNP constructing haplotype has a true contribution to the susceptibility of disease, even though unapparent, haplotype analyses can provide a greater statistical power and are sometimes advantageous over analysis of an individual SNP for detecting an association between alleles and a disease phenotype [53]. We analyzed contributions of different haplotype combinations of 4 *ICAM-1* SNPs (rs3093030, rs5491, rs281432, and rs5498) to the risk of oral cancer and eventually found that the *TAGG* or *TACG* haplotype showed a high risk for OSCC (Table 7). It is possible that the *TAGG* or *TACG* haplotype of *ICAM-1* is in LD with other functional polymorphisms that are responsible for the susceptibility to OSCC.

In conclusion, our results suggest that *ICAM-1* polymorphic rs5498 might be correlated with susceptibility to oral cancer, and combined effects of *ICAM-1* gene polymorphisms with environmental carcinogens significantly increase the risk of developing oral cancer. The *TAGG* or *TACG* haplotype of the 4 *ICAM-1* SNPs (rs3093030, rs5491, rs281432, and rs5498) combined also showed a high-risk association with OSCC. Patients with oral cancer who carry at least 1 T allele of *ICAM-1* rs5491 or 1 G allele of *ICAM-1* rs281432 have a lower risk of developing an advanced clinical stage compared to patients carrying A/A or C/C homozygotes.
